# Optimizing nation-wide locations of dialysis centers: a geographic information system-based approach to improve healthcare accessibility and availability

**DOI:** 10.1186/s13584-025-00704-5

**Published:** 2025-07-15

**Authors:** Hanna Schroeder, Chen Namimi-Halevi, Osnat Luxenburg, Ayelet Grinbaum Arizon, Zach Tagar, Michal Bromberg, Vered H. Eisenberg

**Affiliations:** 1 Policy Planning Department at the Israel Ministry of Health and an instructor in the Henrietta Szold School of Nursing, Hebrew University - Hadassah Faculty of Medicine, Jerusalem, Israel; 3 Medical Technology, Health Information and Research Directorate, Israel Ministry of Health, Jerusalem, Israel; 4 Strategic and Economic Planning Administration at the Israel Ministry of Health, Jerusalem, Israel; 5 BI & GIS Department, Israel Ministry of Health, Jerusalem, Israel; 6 Israel Center for Disease Control, Israel Ministry of Health, Ramat Gan, Israel; 7 Department of Epidemiology and Preventive Medicine, School of Public Health, Gray Faculty of Medical & Health Sciences, Tel Aviv University, Tel Aviv, Israel; 8 Medical Facilities and Equipment Licensing, Medical Technology, Health Information and Research Directorate, Gray School of Public Health, Faculty of Medical & Health Sciences, Ministry of Health, Tel Aviv University, Tel Aviv, Israel

**Keywords:** Hemodialysis, GIS, Healthcare planning, Health policy, Accessibility, Equity, Availability

## Abstract

**Background:**

Accessibility and availability are critical components of quality healthcare, particularly for dialysis patients requiring tri-weekly treatments. Inconveniently placed or oversubscribed dialysis centers contribute to widening healthcare disparities. This study aims to enhance equity in dialysis care by utilizing Geographic Information Systems (GIS) to optimize facility placement through data-driven decision-making.

**Methods:**

This cross-sectional study analyzed national data from 5,961 hemodialysis patients across 76 dialysis centers in Israel. Geographic accessibility was assessed using GIS to measure travel distances between patients’ residences and their treating dialysis centers. For utilization rate, active hemodialysis patient count was compared to estimated maximum capacity for each center. Statistical comparisons across districts were conducted using chi-square, ANOVA, or Kruskal-Wallis tests, with Bonferroni corrections. Findings were visualized using ArcGIS software.

**Results:**

The median travel distance to dialysis centers varied significantly by district (*p* < 0.001), with the longest distance in the North district (10.9 km) and the shortest in the South district (3.4 km). The mean utilization rate was 73.3%, with the highest in the North district (82.5%) and the lowest in the Jerusalem district (64.3%). No significant differences in utilization rates were found between districts (*p* = 0.38.

**Conclusions:**

To our knowledge, this is the first study to apply GIS to national patient-based data for assessing dialysis center accessibility and utilization. Our findings demonstrate how GIS integration with national registries can inform equitable healthcare planning and facility allocation. This approach offers policymakers a scalable, technology-driven strategy to optimize resource distribution, correct healthcare inequities, and improve accessibility for dialysis patients.

**Supplementary Information:**

The online version contains supplementary material available at 10.1186/s13584-025-00704-5.

## Introduction

Dialysis is the predominant vital treatment for the majority of end-stage renal disease (ESRD) patients [1]. The incidence and prevalence of dialysis patients have been increasing both in Israel and globally [2–4]. This trend is characterized by a rising average age of new dialysis patients [4] and a longer life expectancy [5]. As a result, there is a growing demand, and an urgent need to find methods for opening new dialysis centers, that can effectively cater to the expanding and aging dialysis population.

Accessibility plays a crucial role in ensuring optimal care and improved outcomes for dialysis patients [6]. Geographic reachability is measured by the distance from the patients’ address to the attending medical center. Given the frequent nature of hemodialysis treatments, geographical proximity emerges as a fundamental aspect of dialysis care accessibility. Patients living far from dialysis facilities encounter significant obstacles of accessing timely [7] and suitable treatment, due to extended travel durations and potential difficulties in securing reliable transportation [8]. Longer travel distances can have clinical ramifications, including higher mortality rates and a reduced quality of life [9]. 

Availability is measured by having sufficient hours attributed for patient treatment in the facility. It is influenced by an appropriate distribution of dialysis centers and a balance between overburdened and underutilized centers [10]. Overburdened centers may lead to increased wait times, compromised quality of care, and potential burnout among healthcare professionals. Conversely, underutilized centers can result in inefficient resource allocation and limited access to specialized services.

In Israel, hemodialysis centers are licensed by law within the Medical Facilities and Equipment Licensing Division of the Israel Ministry of Health (MoH). All licensed facilities undergo regular and timely inspection visits. License renewal relies on passing the inspection. New dialysis centers undergo inspection before opening for service and must abide by a set of standard criteria.

In the past, healthcare location planning was not based on data-driven decisions and was, therefore, more prone to external factors. As a result, facilities were potentially inconveniently placed or were over-utilized resulting in long waits. This could be especially true for lower socio-economic groups with greater logistic challenges in reaching healthcare facilities [11–13], which can further widen healthcare gaps and disparity of quality and equity.

The aim of this study is to utilize Geographic Information Systems (GIS) to assess spatial gaps in the distribution of dialysis centers across Israel, evaluate the operational burdens on existing centers, and provide recommendations regarding the necessity and optimal geographic placement of additional centers. The goal is to improve equity and equality in accessing dialysis centers.

## Methods

### Study design and participants

This is a national cross-sectional study on all patients undergoing hemodialysis treatment in Israel, as well as on all 76 dialysis centers operating throughout the country’s six districts.

The National Israeli Kidney Replacement Therapy (KRT) Registry, managed by the Israeli Center for Disease Control under the MoH, serves as a comprehensive data repository regarding all patients receiving any form of kidney replacement therapy in Israel [14]. Mandated by the MoH, all nephrology and dialysis units, including community-based clinics and hospital facilities, are required to report data on all new and active KRT patients, as well as any changes in treating center and treatment modality. The reported information includes demographics, the specific dialysis center attended, treatment dates, treatment modalities (hemodialysis or peritoneal dialysis), and primary renal disease diagnosis. Information from the national registry is cross-checked with data provided to the MoH by the insuring health funds for financial compensation purposes. This stringent control mechanism enhances the registry’s data reliability and strengthens its overall validity.

For the purpose of this study, data were obtained regarding all hemodialysis patients as of November 10, 2022. The registration data was cross-referenced with data from the Israeli Ministry of the Interior, enabling the retrieval of precise current home address for the patients.

### Accessibility and availability

Accessibility was determined by measuring travel distances from patients’ current addresses to their attending dialysis center, using GIS. In a supplementary analysis (see Supplementary Table S1 and Figure S1), travel distances to the nearest dialysis center were calculated (“distance to nearest center”). Availability was assessed by measuring the supply and demand of dialysis services, computing the utilization rate of each center. Each center reports the following information to the MoH: number of dialysis stations per center, number of shifts the center operates each week and indication of whether the facility has the capability to accommodate patients with substantial physical limitations who require hemodialysis administration in the supine position.

The utilization (U) of each center, as of November 10, 2022, was calculated by dividing the number of active dialysis patients (AP) treated in the center by the number of potential patients (PP) the facility can treat at any given time. The number of PP was computed by multiplying the number of dialysis stations (NS) in each center by the number of shifts the center operates each week (S) and then divided by 3, the average number of weekly treatments a patient receives. For example, if a dialysis center has 12 stations and operates 12 shifts per week, it can treat 48 potential patients. If, according to the national registry, the center treats 40 active patients, then it has a utilization rate of 83%.

$$\:PP=\left(NS*S\right)/3$$

$$\:U=AP/PP$$

### Mapping

In addition, geographic analysis was conducted using ArcGIS Pro 3.3 software (Environmental Systems Research Institute), allowing the integration of multiple variables into real-world geographic mapping. The variables included in the assessment were: (1) the patient’s home address; (2) the dialysis center address; (3) the road travel distance from the patient’s home address to the attending dialysis center; (4) the utilization (U) of each center; (5) an indication of whether the center can treat patients that require treatment in the supine position. The inclusion of this last variable is important for future dialysis center planning due to the aging of dialysis patients.

### Geographic, sociodemographic, and treatment-related variables

The following variables were extracted from the Israeli National KRT Registry, based on point prevalence data at the date of data extraction (November 10, 2022): age (as a continuous variable and by the following age groups: 0–19, 20–44, 45–54, 55–64, 65–74, 75–84, 85 + years); sex (male, female); population group (Arabs vs. Jews and others, where “others” refers to individuals not classified as Jews or Arabs, such as non-Arab Christians, individuals without religious affiliation, and other minority groups); treatment setting (hospital-based vs. privately operated community-based dialysis units); and primary kidney disease (diabetes mellitus, hypertension, glomerulonephritis, polycystic kidney disease, pyelonephritis, renal vascular disease, other, unknown, or missing).

Residential district was derived from address data obtained through linkage with the Ministry of Interior. Socioeconomic status (SES) was assigned based on the statistical geographic area of residence using the Points Location Intelligence (PLI) Index. This index provides a neighborhood-level classification and ranking of the residential population’s SES. It is based on the Israeli Central Bureau of Statistics (CBS) socioeconomic cluster classification, which incorporates demographic composition, educational attainment, living conditions, employment status, and pension data. The index is continuously updated to reflect changes in population composition, the development of new neighborhoods, and sector-specific characteristics not fully captured by the CBS classification. The final PLI index ranges from 1 (lowest SES) to 10 (highest SES). For the purposes of this analysis, SES was categorized into three groups: low [1–3], medium [4–6], and high [7–10].

### Statistical measures

Data were analyzed using SPSS software, version 29. Categorical variables were described as frequencies (percentages) and quantitative variables as either means (standard deviations) or medians (first and third quartiles), based on their distribution.

In the bivariate analyses, the associations between the independent variable (district of residence) and the dependent variables (travel distances and utilization of dialysis centers) were examined using the chi-square test, or either the ANOVA test or the Kruskal-Wallis test, depending on the dependent variable’s distribution. Post-hoc analysis of pairwise comparisons with Bonferroni correction was applied for multiple comparisons.

To remove upper limit outliers from the accessibility analysis, the interquartile range (IQR) was calculated. Cases where the travel distance exceeded the third quartile (Q3) by more than 6 times the IQR were excluded from the analysis. This method excludes participants whose travel distance is suspected to not accurately reflect the true distance traveled, likely due to incorrect address information in the Ministry of Interior’s records for various reasons. A lower limit was not applied to exclude outliers, as some patients are treated at the facility where they reside such as long term care facilities for older adults with dialysis care, resulting in a distance of 0 km. A sensitivity analysis was conducted which included all patients for whom travel distance to t he dialysis center could be obtained, without excluding outliers. All tests were two-tailed, with a significance level set at 0.05.

### Ethics approval

#### Ethical approval

Ethical approval for this study was exempted by the National Ethical Committee for Human Medical Research of the Israeli Ministry of Health. The committee determined that the survey was integral to the Ministry’s official activities.

## Results

### Participant characteristics

As of November 10, 2022, there were a total of 6,230 hemodialysis patients (point prevalence) from six districts (487 municipalities) across Israel. For 200 of them (3.2%), travel distance could not be obtained due to technical reasons.

Before removing outliers, the IQR for travel distance was 8.9 km, with the third quartile at 11.8 km. Based on the outlier exclusion rule, participants whose travel distances exceeded six times the IQR above the third quartile were excluded. Therefore, 69 participants, for whom travel distance exceeded 65.1 km, were removed from the main analysis (representing 1.1% of the hemodialysis patients with recorded distances). The proportion of excluded participants was higher in two districts: South (3.3%, *n* = 32) and Haifa (1.2%, *n* = 11), but the actual number of patients excluded per district remained low.

After excluding outliers, the primary analysis included 5,961 hemodialysis patients. Their mean age was 67.9 ± 14.1 years, and the majority (63%) were male. About 30% were 65–74 years old, and approximately 73% were of low to medium SES. Half of the patients were treated in facilities located inside hospitals, and the most common primary renal disease was due to diabetes mellitus (Table 1). Approximately a quarter of the patients resided in the Central district, and another 20% in the Northern district. As shown in Table 1, significant differences were found between the districts regarding the distribution of patients by age group, gender, population group, SES, primary renal disease, and treatment location (hospital vs. community). Arab patients accounted for 61.1% of the dialysis population in the North district, compared to 2.8% in the Tel Aviv district and 12.1% in the Center district. Similarly, the proportion of patients with low SES was approximately 51% in the North district, compared to 4.8% in both the Tel Aviv and Center districts. Patients in the North district were also younger on average, with 43.3% under the age of 65—the highest proportion among all districts and above the overall national percentage of 34.2%.

**Table 1:** Characteristics of prevalent Hemodialysis patients in Israel (as of November 10, 2022)

Variable	All (*n* = 5961)	North (*n* = 1160)	Haifa (*n* = 925)	Center (*n* = 1453)	Tel Aviv (*n* = 878)	Jerusalem (*n* = 596)	South (*n* = 949)	*P*-value
Age, years, mean ± SD	67.94 ± 14.09	65.37 ± 14.26	67.92 ± 14.38	68.96 ± 13.35	72.16 ± 12.09	65.00 ± 15.94	67.5 ± 14.06	< 0.001
Age groups, n (%):								< 0.001
0–19	23 (0.4)	8 (0.7)	1 (0.1)	1 (0.1)	1 (0.1)	7 (1.2)	5 (0.5)	
20–44	375 (6.3)	79 (6.8)	69 (7.5)	74 (5.1)	25 (2.9)	59 (9.9)	69 (7.3)	
45–54	532 (8.9)	139 (12.0)	77 (8.3)	131 (9.0)	52 (5.9)	61 (10.2)	72 (7.6)	
55–64	1111 (18.6)	276 (23.8)	170 (18.4)	272 (18.7)	109 (12.4)	113 (19.0)	171 (18.0)	
65–74	1812 (30.4)	336 (29.0)	285 (30.8)	420 (28.9)	278 (31.7)	180 (30.2)	313 (33.0)	
75–84	1539 (25.8)	251 (21.6)	219 (23.7)	409 (28.1)	277 (31.6)	136 (22.8)	247 (26.0)	
85+	569 (9.6)	71 (6.1)	104 (11.2)	146 (10.1)	136 (15.5)	40 (6.7)	72 (7.6)	
Sex, n (%) males	3753 (63.0)	673 (58.0)	599 (64.8)	976 (67.2)	560 (63.8)	374 (62.8)	571 (60.2)	< 0.001
Population group, n (%) Jews and others	4330 (72.6)	451 (38.9)	680 (73.5)	1277 (87.9)	853 (97.2)	268 (45.0)	801 (84.4)	< 0.001
Socioeconomic status, n (%)								
Low	1606 (27.3)	577 (50.9)	214 (23.3)	144 (4.8)	42 (4.8)	307 (53.6)	322 (34.7)	< 0.001
Medium	2725 (46.4)	491 (43.4)	514 (55.9)	667 (46.2)	425 (48.5)	179 (31.2)	449 (48.4)	
High	1543 (26.3)	65 (5.7)	192 (20.9)	634 (43.9)	409 (46.7)	87 (15.2)	156 (16.8)	
Treatment location, n (%) hospital	2952 (49.5)	641 (55.3)	365 (39.5)	866 (59.6)	555 (63.2)	253 (42.5)	272 (28.7)	< 0.001
Primary renal disease, n (%)								< 0.001
Diabetes mellitus	2701 (45.3)	576 (49.7)	439 (47.5)	616 (42.4)	378 (43.0)	297 (49.8)	395 (41.6)	
Hypertension	693 (11.6)	132 (11.4)	66 (7.1)	205 (14.1)	107 (12.2)	73 (12.2)	110 (11.6)	
Glomerulonephritis	449 (7.5)	80 (6.9)	82 (8.9)	111 (7.6)	57 (6.5)	46 (7.7)	73 (7.7)	
Polycystic kidney disease	195 (3.3)	40 (3.5)	24 (2.6)	47 (3.2)	34 (3.9)	22 (3.7)	28 (3.0)	
Pyelonephritis	135 (2.3)	20 (1.7)	21 (2.3)	38 (2.6)	22 (2.5)	9 (1.5)	25 (2.6)	
Renal vascular disease	77 (1.3)	12 (1.0)	9 (1.0)	16 (1.1)	28 (3.2)	8 (1.3)	4 (0.4)	
Miscellaneous	597 (10.0)	105 (9.1)	70 (7.6)	158 (10.9)	102 (11.6)	69 (11.6)	93 (9.8)	
Unknown	833 (14.0)	147 (12.7)	192 (20.8)	164 (11.3)	107 (12.2)	48 (8.1)	175 (18.4)	
Missing	281 (4.7)	48 (4.1)	22 (2.4)	98 (6.7)	43 (4.9)	24 (4.0)	46 (4.8)	

### Accessibility

**Fig. 1:**
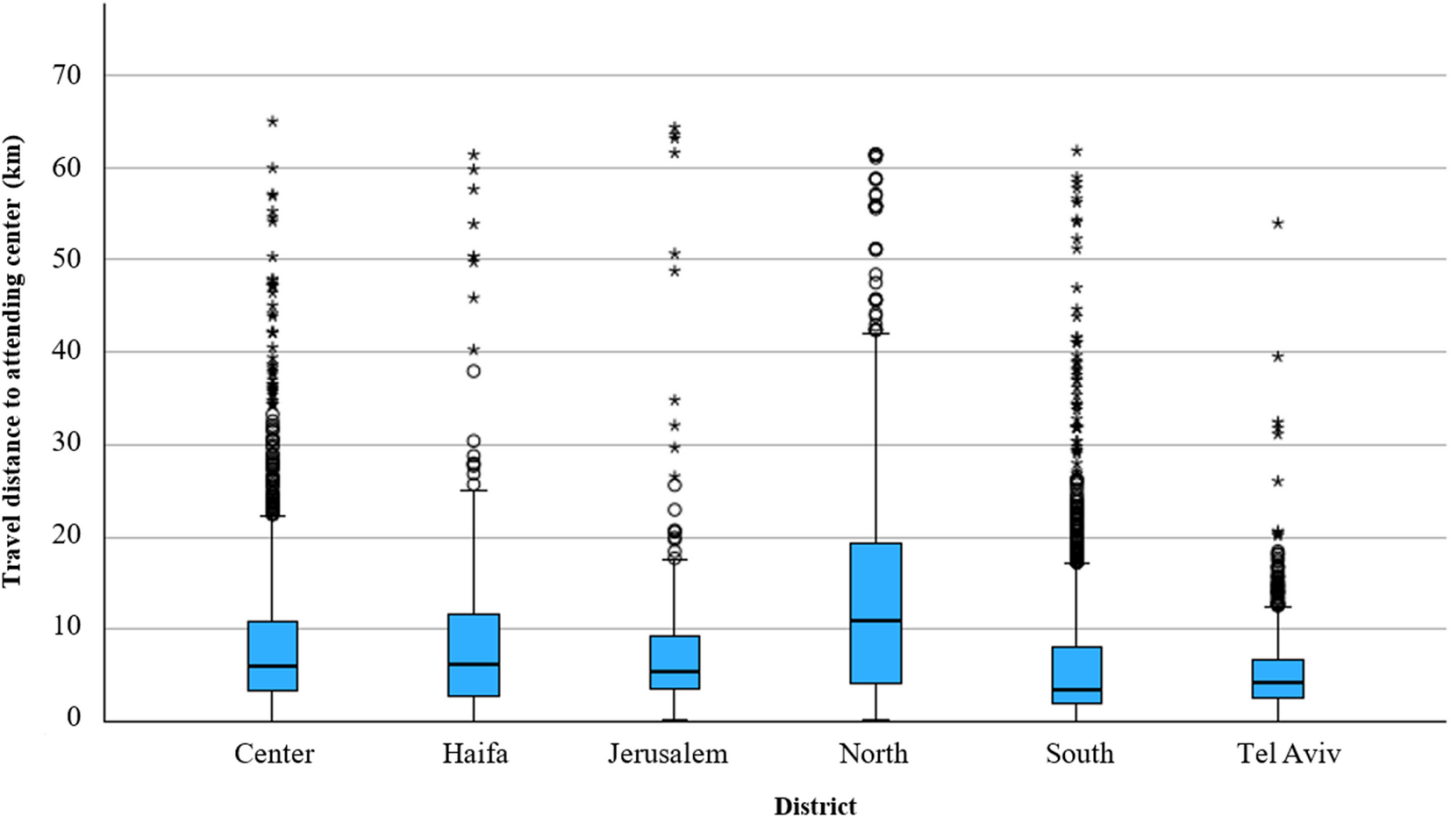
Travel distances to attending dialysis centers by districts. Circles represent moderate high outliers (values between 1.5 and 3 times the interquartile range [IQR] above the third quartile), while asterisks indicate extreme high outliers (values exceeding 3 times the IQR above the third quartile)

The median travel distance was 5.3 km, with a range of 0–65 km. The longest median travel distance was in the North district (10.9 km), while the shortest median travel distance was in the South district (3.4 km) followed by the Tel-Aviv district (4.2 km) (Table 2; Fig. 1). Travel distances were found to be significantly different between the districts (*p* < 0.001).

**Table 2:** Accessibility and utilization of attending dialysis centers by districts

	*Accessibility* ^*a*^					*Utilization* ^*b*^				
District	N	Median Travel Distance (Q1, Q3) (km)	Minimum Distance (km)	Maximum Distance (km)	Range (km)	Number of Centers	Mean % of utilization ± SD	Minimum utilization (%)	Maximum utilization (%)	Range (%)
All	5961	5.3 (2.9, 11.8)	0.0	65.0	65.0	76	73.3 ± 21.1	12.3	117.3	105.0
North	1160	10.9 (4.2, 19.3)	0.2	61.4	61.2	12	82.5 ± 25.6	47.6	117.3	69.7
Haifa	925	6.3 (2.8, 11.7)	0.0	61.4	61.4	12	79.1 ± 24.4	40.2	103.6	63.4
Center	1453	6.1 (3.3, 10.9)	0.0	65.0	65.0	22	71.6 ± 19.3	23.8	100.0	76.2
Tel Aviv	878	4.2 (2.7, 6.6)	0.1	54.0	53.9	11	71.0 ± 15.1	41.9	94.0	52.1
Jerusalem	596	5.5 (3.6, 9.3)	0.2	64.4	64.2	9	64.3 ± 25.5	12.3	90.8	78.5
South	949	3.4 (2.0, 8.1)	0.0	61.8	61.8	10	70.0 ± 14.5	47.6	92.6	45.0

^a^The difference in accessibility (travel distances) between the districts is statistically significant (*p* < 0.001)

^b^The difference in facility utilization rates is not statistically significant (*p* = 0.38)

**Fig. 2:**
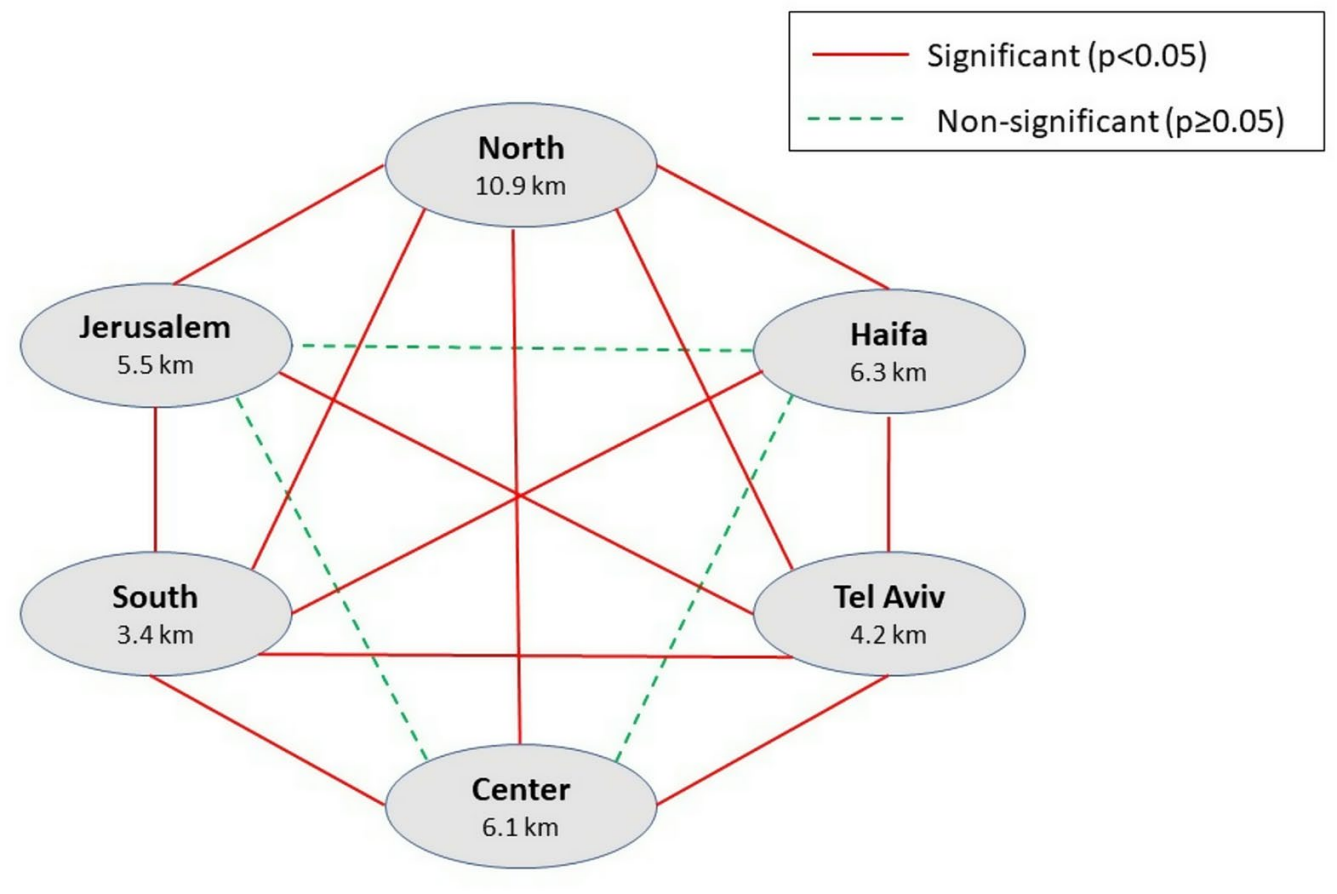
Pairwise comparison of median travel distances to attending dialysis centers by district (Q3 + 6*IQR)

Figure 2 presents a pairwise comparison between districts of travel distances to attending dialysis centers, highlighting that two districts show statistically significant differences compared to all other districts: the North district, with a notably higher median travel distance, and the South district, with a significantly lower median travel distance.

The sensitivity analysis, that included outliers in travel distances, demonstrated similar results regarding significant differences in travel distances between districts with one exception: no significant difference was found in the median of travel distances between the South and Tel-Aviv districts.

In the supplemental analysis (see Supplementary Table S1 and Figure S1), the median distance to the nearest dialysis center was 3.3 km, ranging from 0 to 56.7 km. As in the main analysis, the longest median distance was observed in the North district (6.5 km). The shortest distance was in the Tel Aviv district (2.4 km), followed by the South district (2.7 km). Statistically significant differences in travel distances to the nearest center were observed in all pairwise comparisons (*p* < 0.05), except for the following pairs: South–Center, Center–Haifa, Center–Jerusalem, and Haifa–Jerusalem.

### Utilization of dialysis centers

**Fig. 3:**
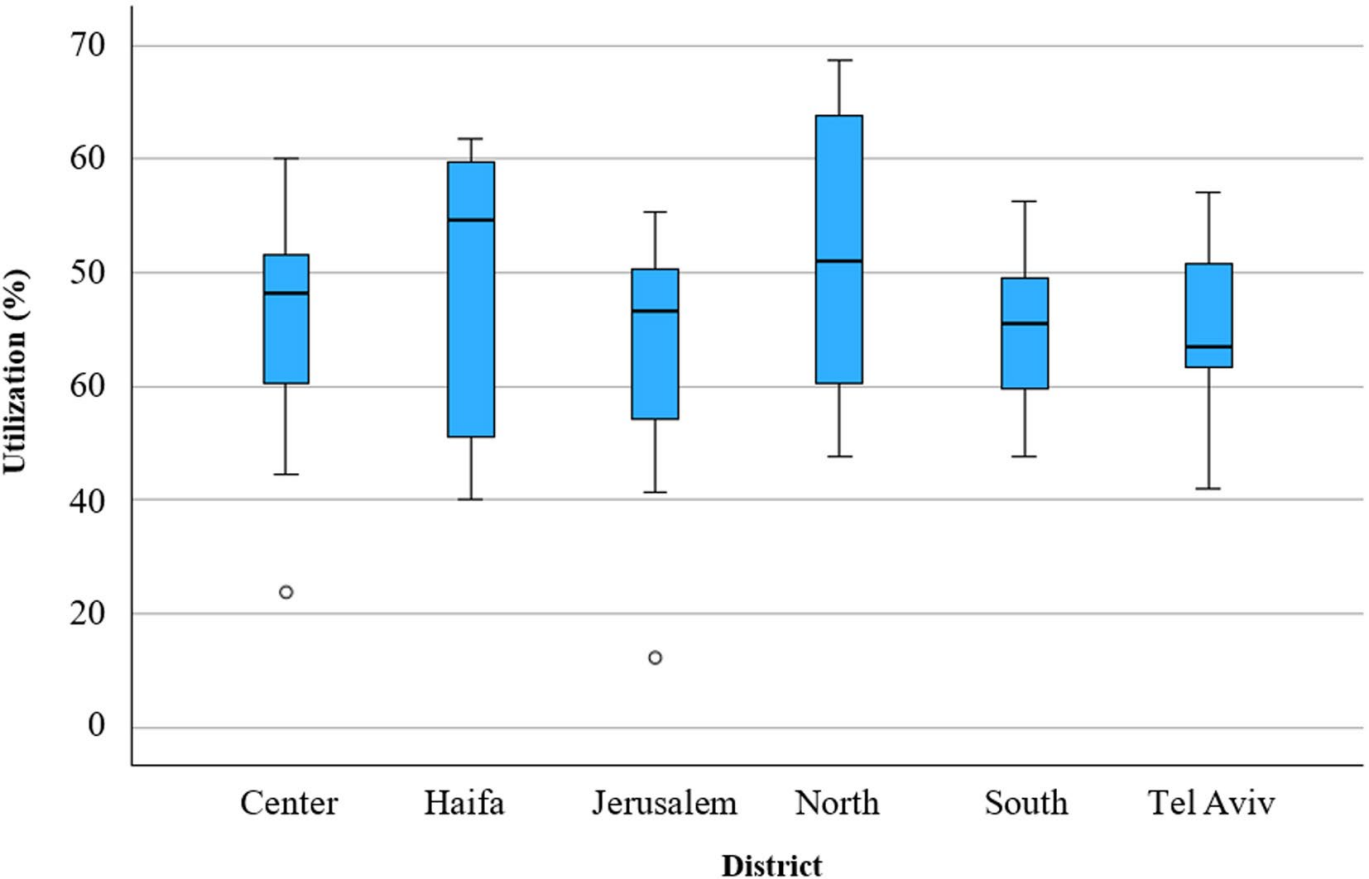
Utilization rate of dialysis centers by district. Circles represent moderate high outliers (values between 1.5 and 3 times the interquartile range [IQR] above the third quartile)

**Fig. 4:**
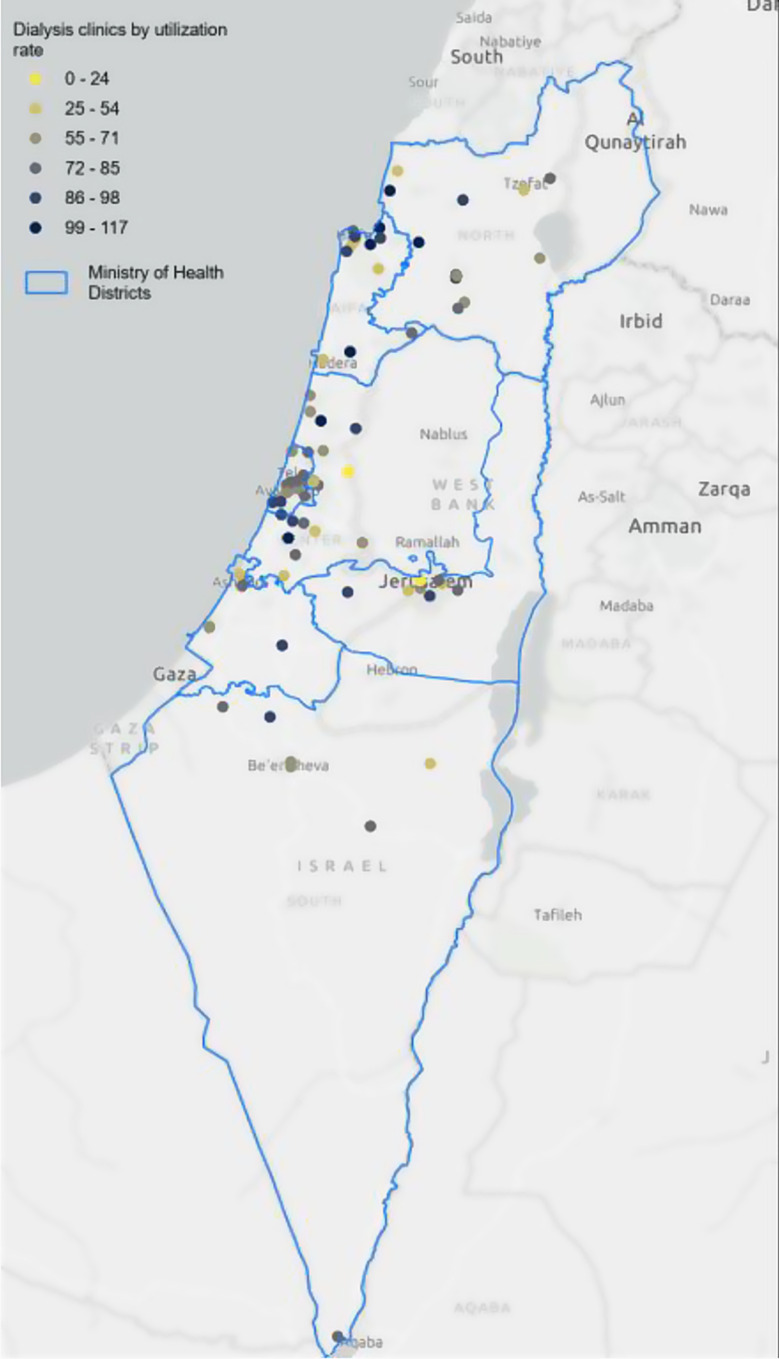
Map of utilization rate of dialysis centers across Israel

The mean utilization rate in all 76 dialysis centers across Israel was 73.3%, with a range of 12.3–117.3% (see Table 2 and Figs. 3 and 4). The highest mean utilization rate was documented in the North District, at 82.5%, with the highest maximum rate of 117.3%. The lowest mean rate was noted in the Jerusalem district, at 64.3%, with the lowest maximum rate of 90.8%. No significant differences in mean utilization rates were found between the districts (*p* = 0.38) (Table 2).

### Scheduling of dialysis shifts

The scheduling of dialysis shifts is determined by the requirement for patients to receive treatment every other day. The day is usually divided into three shifts, morning, noon, and afternoon. Consequently, the standard distribution followed either Sunday-Tuesday-Thursday pattern or a Monday-Wednesday-Friday pattern, where a third shift is feasible on Fridays. The implementation of Friday third shifts is predominantly viable in Arab communities rather than Jewish communities due to religious observances. For this reason, the capacity to incorporate additional third shifts is not universally feasible across all demographic contexts.

### Accommodations to patients that require Hemodialysis administration in the supine position

Overall, A total of 37 (47%) dialysis centers were accustomed for providing treatment for patients with significant functional limitations requiring supine-position dialysis therapy, 4 (11%) in the North district, 6 (16%) each in Haifa, and the South districts, 10 (27%) in the Center district, 10 (27%) in the Jerusalem district and 7 (19%) in the Tel Aviv district. These centers were primarily located in nursing homes (22%) and hospitals (47%).

## Discussion

### Main findings

This study highlights substantial variations in ESRD patients’ travel distances to dialysis centers and in the utilization rate of each center. Compared to other districts, patients residing in the North district travel greater distances to both their treating dialysis centers and the nearest available centers. Although not statistically significant, the centers in this district are, on average, more burdened. These disparities in healthcare distribution have a potential to expand due to the ongoing aging of the total population and the higher rates of diabetes mellitus, a major risk factor for ESRD [15], in the North District [16]. 

The findings indicating that travel distances were longer in the North district—both when calculated based on the actual treating facility and when based on the nearest available facility—reinforce the interpretation that this region requires targeted intervention to improve geographic accessibility. Even under an ideal scenario in which all dialysis centers possess the capacity to treat any patient, regardless of facility type (hospital-based or community-based), and without constraints such as budgetary approvals from health funds, this district would remain at a disadvantage in terms of dialysis infrastructure distribution.

It is noteworthy that in the North district—where travel distances were longer—the patient population includes a relatively high proportion of individuals from low socioeconomic backgrounds and of Arab ethnicity. In addition, the finding that patients in this district are younger on average may also suggest that older individuals residing in this high travel-burden district face difficulties accessing treatment due to geographic barriers and are therefore underrepresented among patients in this region. These observations may point to underlying health disparities and highlight the need for further investigation in future studies. Improving geographic accessibility for socioeconomically disadvantaged populations is particularly important, as the burden of distance may impose greater challenges on these groups compared to more advantaged populations—for example, in relation to private car ownership, which facilitates access to treatment.

Our research demonstrates how combining a national registry of dialysis patients with dialysis clinics’ locations and utilization rates using new and advanced computing technologies, such as GIS tools, is useful for defining health care needs and discrepancies nationwide. While mapping the patients and their treating centers, we also took under consideration additional variables, such as an indication if the facilities are accommodated for patients that require hemodialysis administration in the supine position, due to the aging of the population. Various possible solutions exist to improve dialysis centers’ accessibility and availability. This study can enhance the ability to choose the best solutions based on the data collected per dialysis center, among these are: increasing the number of shifts, adding dialysis stations in existing centers, building new dialysis centers, etc. There is also a possibility of home-hemodialysis, which is not widely spread to date. GIS, as proposed in this study, can, therefore, be used to proactively and centrally plan national dialysis care that will reflect both the patients’ needs and the available resources, and subsequently will improve equity and equality in healthcare planning and allocation.

The use of maps in healthcare dates back to the 19th century when the epidemiologist John Snow mapped Cholera cases in London [17]. In recent years, there has been an improvement in GIS tools and an expansion in their use in healthcare. Maps had been used prominently in the COVID-19 outbreaks [18]. GIS tools were also used for monitoring the effect of environmental factors on health [19–21], computing geographical access to healthcare facilities [10, 22–25], assessing the accessibility to healthcare services in different transportation modes [26], measurement of catchment area of healthcare facilities [27–29], evaluating areas at high-risk for health emergency events such as strokes [30], and also to identify the optimal routes of pre-hospital services [31] and locations for automated external defibrillators [32]. There is even a study [33] that described using GIS tools for measuring accessibility to dialysis centers on a small scale and spatial accessibility models that had taken the distributions of older adults into account [32, 34]. 

Nevertheless, there are still gaps in the assimilation of GIS tools for nationwide healthcare services planning [35–38]. For example, many of the studies that addressed healthcare accessibility assessed access of populations to healthcare within a defined catchment area [39]. Moreover, these studies did not take into account the specific center where the patient was treated and/or used national individual patient information regarding the travel distances to the treating centers. They also did not consider the exact supply and demand of each center. For instance, Yant et al. [40], who used this method among dialysis patients in the city of Chicago, USA, stressed the importance of having patient-based data that can enable accurate measuring of a facility’s capacity. Furthermore, three systematic reviews [38, 41, 42] of GIS research stressed the shortage of studies that combine individual patients’ information (non-spatial factors) and sophisticated GIS tools (Spatial factors) to improve equity and the limited ability to accurately measure supply and demand.

Contrary to the current literature, this study enabled, on a national level, a measurement of patients’ travel distances to their treating dialysis centers and supply vs. demand of all centers. The information regarding the demand of dialysis patients was obtained from a national population-based registry. The information for supply was obtained from the license information of all dialysis centers that contains individual data on number of operating dialysis machines and shifts each week, thus reflecting the true supply. To the best of our knowledge, this is the first study to implement a robust evidence-based approach of combining several measures using GIS for planning national healthcare facilities, while ensuring sufficient geographic coverage for patients in Israel, and avoiding both excessive workload and under-utilization. Although we focused our study on ESRD patients, since they particularly need repeated weekly treatments for a substantial period of time, similar models can be used for other healthcare services.

This study had several limitations. First, although real distances from home address to treating dialysis clinic were calculated taking into consideration the current road infrastructure, we were unable to account for actual transportation times, that may vary throughout the day and across areas, thereby influencing the accessibility of the dialysis centers. Second, the patients’ addresses were obtained from the Israeli Ministry of the Interior database, which may not be a 100% accurate. To eliminate this bias, we removed upper outliers of travel distances from the analysis. Additionally, information on the functional status of dialysis patients, overall and by district, was not available, limiting the ability to interpret the degree of alignment between the proportion of centers that can accommodate patients with significant functional limitations and the potential need nationwide and in each district. As a future goal, it is recommended that such data be documented at the national level to support more tailored planning efforts.

## Conclusion

In conclusion, this nationwide project has the potential to enhance the accessibility and availability of healthcare facilities and services. The use of advanced technology combined with national patient-based data can significantly improve the quality of care by ensuring both equity and equality. This approach offers a scalable model that can be implemented in other countries and across additional healthcare fields to address systemic disparities effectively.

## Electronic supplementary material

Below is the link to the electronic supplementary material.

Supplementary Material 1

## Data Availability

No datasets were generated or analysed during the current study.

## Abbreviations

CBS: Central Bureau of Statistics
GIS: Geographic Information System
KRT: Kidney Replacement Therapy
PLI: Points Location Intelligence
SES: Socioeconomic Status
ESRD: End Stage Renal Disease
MoH: Ministry of Health
IQR: Interquartile Range
