# Supplementary material for: Optimizing nation-wide locations of dialysis centers: a geographic information system-based approach to improve healthcare accessibility and availability
Source: Isr J Health Policy Res. 2025 Jul 15;14:40. doi: 10.1186/s13584-025-00704-5 (PMC12261728; doi:10.1186/s13584-025-00704-5)
Supplement: Supplementary file 1 — Supplementary Material 1 [file 13584_2025_704_MOESM1_ESM.docx]

Supplementary

**Table 1: Accessibility of nearest dialysis centers by districts**

|  | **Accessibility^a^** | | | | |
| --- | --- | --- | --- | --- | --- |
| District | N | Median Travel Distance (Q1, Q3) (km) | Minimum Distance (km) | Maximum Distance (km) | Range (km) |
| All | 5961 | 3.3 (2.1, 5.9) | 0.0 | 56.7 | 56.7 |
| North | 1160 | 6.5 (3.4, 11.9) | 0.0 | 48.9 | 48.9 |
| Haifa | 925 | 3.1 (2.0, 5.6) | 0.3 | 21.3 | 21.0 |
| Center | 1453 | 3.2 (2.3, 4.9) | 0.4 | 56.7 | 56.3 |
| Tel Aviv | 878 | 2.4 (1.7, 3.2) | 0.0 | 10.0 | 10.0 |
| Jerusalem | 596 | 3.5 (2.4, 5.5) | 0.3 | 18.8 | 18.5 |
| South | 949 | 2.7 (1.8, 5.9) | 0.0 | 46.9 | 46.9 |

^a^The difference in accessibility (travel distances) between the districts is statistically significant (p<.001).

**Figure 1: Travel distances to nearest dialysis center by districts**


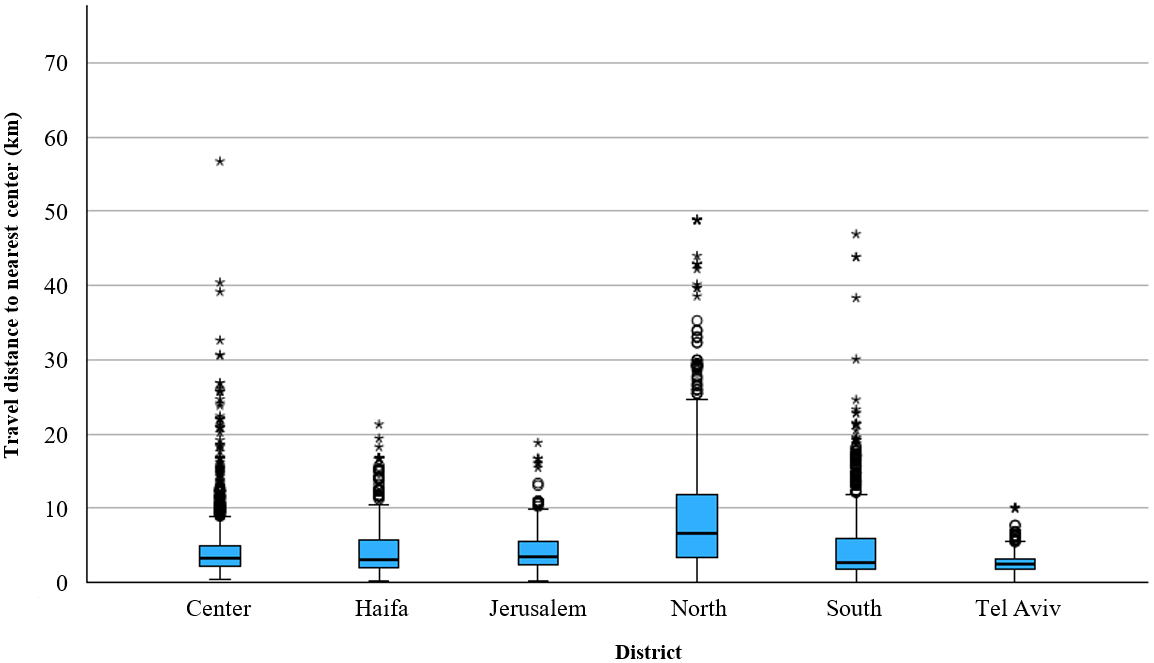


Circles represent moderate high outliers (values between 1.5 and 3 times the interquartile range [IQR] above the third quartile), while asterisks indicate extreme high outliers (values exceeding 3 times the IQR above the third quartile).
